# Paraplegia in Connection with Potts' Disease

**Published:** 1911-04-29

**Authors:** Donald Armour

**Affiliations:** Surgeon to the Hospital.


### HOSPITAL CLINICS.

### PARAPLEGIA IN CONNECTION WITH POTTS' DISEASE.

By DONALD ARMOUR, F.R.C.S., Surgeon to the Hospital.

Delivered at the National Hospital for the Paralysed and Epileptic, Queen's Square, W.C.

Ladies and Gentlemen,—I propose to discuss with you this afternoon paraplegia in connection with Potts' disease; or spondylitis tuberculosa, as it is sometimes called. Paraplegia, like angular curvature in Potts' disease, is a reproach not only to our methods of diagnosis but also to our methods of treatment. It is a complication which should not occur.

### Angular Curvature.

Angular curvature is seen so frequently in Potts' disease that the term angular curvature has come to be used almost synonymously with caries of the spine, as though the angular curvature was a necessity for the diagnosis of Potts' disease; whereas, of course, it is a reproach either to our methods of diagnosis or to our means of treatment. In the same way, I am afraid, we think so little in cases of Potts' disease of the possibility and the frequency of the occurrence of paraplegia, that we never realise its onset until it is definitely and obviously established as, possibly, a complete spastic paraplegia. But it is one of those complications which one ought to be on the look out for from the very earliest onset of the disease, because it is well known—and our experience here bears that out—that in the large majority of cases the onset of paraplegia occurs more frequently in those cases in which there is no angular curvature than the reverse. As far as my recollection serves me, the majority of cases we see here are those in which the patients have little or no bony deformity associated with the paraplegia. So it should be remembered that the onset of paraplegia bears no relation to either the onset or the degree of the deformity. And the reverse is true, as I shall show you presently on the screen: there can be a very large amount of angular deformity and even bone destruction and abscess formation, without involvement of the cord or of nerves at all.

### Causes of Paraplegia.

Now, as to the causes of paraplegia in association with Potts' disease. Time, of course, will not allow me to go into the whole question of pathology, diagnosis, and treatment, so I shall confine myself to paraplegia itself. The causes of paraplegia in Potts' disease may be put under three heads. First, there is direct bony pressure; second, the displacement of a portion of bone, or pressure due to a sequestrum; thirdly, pressure due to caseous material, tuberculoma, tuberculous granuloma, as it is sometimes called, invading the spinal canal and causing not only mechanical pressure on the roots and cord, but setting up by infection tuberculous pachymeningitis. I have given you those causes in the order of least frequency. I may say in passing that a tuberculous myelitis has been described, and possibly it occurs, but it must be very rare; that is to say, a myelitis primarily tuberculous or one which is secondary to pachymeningitis, the latter giving rise to infection of the underlying cord, or any other cases in which primary tuberculous myelitis affects the cord itself. The paraplegia in Potts' disease caused by direct bony pressure is very rare. It is more frequently due to pressure caused by the separation of some of the diseased bone in the form of a sequestrum. But even these constitute only about 2 per cent. of the cases. The vast majority of cases are due to pressure from tuberculous material which, breaking through from the bodies of the vertebræ and through the posterior common ligament, invades the spinal canal, causing either direct pressure or, usually, a combination of direct pressure and pressure due to infective tuberculous pachymeningitis, causing immense thickening of the dura surrounding the nerve roots and the spinal cord itself. Sometimes this caseous material goes on to the formation of tuberculous pus, such as we see in other situations; but more often it is due to the thick caseous material which we describe as tubercular granulation tissue. The actual lesion due to the pressure is usually limited to one or two segments of the cord; less frequently more, not more than half an inch of cord being affected by pressure. But the tuberculous pachymeningitis may spread very much further than the actual cord lesion. I shall show you a specimen shortly in which the tuberculous pachymeningitis at the seat of the cord lesion is very slight indeed. But far removed from the cord lesion you will see the extent to which this tuberculous pachymeningitis has gone on.

### Some of the Main Symptoms.

Before discussing the pathology further, I want to run over very rapidly with you the symptoms which one may expect to find in cases of paraplegia associated with Potts' disease. I have not thought it necessary to bring down a patient, because those of you who were here last Friday, at Dr. Ormerod's lecture, saw a very typical case of spastic paraplegia due to Potts' disease. The first symptoms are liable to be overlooked, and as a rule they are very slight, commencing probably very gradually, though possibly the onset may be quite sudden, like that of a fractured dislocation where a sequestrum has been separated. More often the onset is gradual, and the early symptoms are completely overshadowed or masked by the symptoms of the primary disease. There are girdle pains, so-called stomach-ache, where the disease is in the dorsal region, the child tires or trips easily when running about, or shows a disinclination to run about, or rests frequently when at play; these are some of the earliest symptoms of involvement of the roots or the cord. Perhaps one of the earliest symptoms to be noticed is the frequency with which the child seems to catch its toe when running, or to rest leaning on a chair for a little before going further. It is not until something occurs which leads to a thorough examination being made that the neurological symptoms are made clear. The neurological symptoms in Potts' disease concern both the spinal roots and the spinal cord. The root symptoms are usually the first; sometimes they both appear simultaneously. Less often the cord symptoms appear first. Where there is involvement of roots the symptoms will obviously depend upon the fact that certain roots are involved. In the dorsal region, where the disease is worst of all, the radiating pain may be unilateral, but usually it is bilateral, and in the costal nerves around, and is then often mistaken for so-called intercostal neuralgia. Then we have varying degrees of anæsthesia in the course of the affected roots; possibly herpes zoster occurring as a result of infection of the nerve root. If the dorsal roots lower down, from the seventh to the twelfth, are affected, then one looks for some alteration in the abdominal reflexes, some alteration in the condition of the abdominal muscles, either irritative or paralytic. If the roots coming off the cervical enlargement —that is to say, the roots of the brachial plexus—are affected, you must look for some irritative or paralytic phenomenon in connection with the nerves of the brachial plexus. Of those the eighth cervical and first dorsal will give you pain and sensory disturbances in the area supplied by the ulnar nerve, so that atrophy and paralysis of the small muscles of the hand, and possibly the oculo-pupillary symptoms, are due to involvement of the first and second dorsal nerve. One has frequently wondered why, in this region, these oculo-pupillary symptoms were so often absent. Crouch has shown recently that these symptoms are more frequently absent when the cord is involved than when the nerve roots alone are involved.

### Sensory Symptoms.

Higher up, in the fifth and sixth, you get paralysis affecting the deltoids and supinator longus, and there is an affection of the arm and forearm on that side. So that wherever the roots are affected by the disease, so you will get corresponding symptoms at the periphery due to involvement of these roots. When you come to the symptoms due to cord involvement, then again there is a differing syndrome, depending on the position of the disease. Most frequent in the dorsal region you get the well-known spastic paraplegia, with increased reflexes, and Babinski's and Oppenheim's sign. There are various degrees of paræsthesia, usually a zone of hyperæsthesia overtopping it. Fecler has shown that tactile sensation and sensation to heat and cold are more frequently affected than is pain sensation, and that the sense of position is affected least frequently of all. In association with the pain loss you can get girdle sensation. Late in the disease, as a rule, you find disturbance of the functions of the bladder and rectum, and trophic conditions, as seen in the occurrence of bedsores. These latter are seen only in cases which are very acute, and in which the cord is greatly involved, or in long-standing cases in which the disease has spread. Where the cord is affected in the lower cervical region, that is to say, at the cervical enlargement, there is spastic paralysis, with all its associated signs, of the lower extremity, with practically atrophic paralysis of the upper extremity. The symptoms due to involvement of the uppermost cervical region of the cord—the occipito-atlantoid, involving the articulation between the skull and the atlas—are numerous and often misleading, because they do occur also in other conditions. One of the earliest symptoms of neurological involvement in this region is occipital neuralgia, either unilateral or bilateral, accompanied or followed by anæsthesia over the area of the great occipital nerve, passing round the scalp and upper portions of the cervical nerves. In addition, there may be affection of the spinal accessory and hypoglossal nerves, so that cases are reported in which unilateral, or even bilateral, atrophy of the tongue has occurred due to involvement in Potts' disease. Pressure may give rise to bulbar symptoms; there may be bulbar palsy and difficulty in swallowing, in articulation, and so on.

### Effects are Due to Pressure.

Turning now to the pathology of paraplegia in Potts' disease, we learn that a number of factors have been stated to account for the different conditions found. No doubt in the majority of cases all the three factors I shall mention take part in the production of the condition: (1) Pressure on blood vessels, causing ischæmia of the cord, and followed by necrosis; (2) Pressure on lymphatics, causing lymphatic stasis and œdema; (3) Direct mechanical pressure. I will not mention myelitis, because it is so rare and it would tend to complicate matters. As a result of the combination of these causes we get the final results seen in these cases, the final stage being that of sclerosis or interstitial myelitis.

You will see on the screen an epidiascope picture taken from a very prolonged case which we had in this hospital, showing very well the condition of pachymeningitis. It is a portion of the body of a vertebra in a condition of tuberculous, caseous disease. You see the dura, which has been lifted off; it was nowhere adherent to the posterior common ligament. In spite of all that disease, and in spite of the close proximity of the posterior common ligament—and on comparing it with the dura higher up you will realise what I meant when I said that far from the site of the disease you may get a spread of this tuberculous pachymeningitis giving rise to thickening of the dura—in spite of all this the patient recovered from the paraplegia. The thickening of the dura is far more marked away from the site of the abscess than it is near it.

The next three slides are taken from different cases to show the varying conditions which I have described. One presents some thickening of the membranes, with œdema of the nerve structures, both axis cylinders and medullary sheaths being swollen in these cases, causing œdema of the whole cord. The next picture shows the other process, vascular constriction with necrosis. Here again you see enormously thickened membranes round the cord, and subsequent necrosis in the cord itself. In the third slide we see the cord practically reduced to scar tissue: enormously thickened membranes, causing constriction, and degeneration taking place. This last slide was taken from a long-standing case, which was originally one of œdema.

*(To be continued.)*

